# Acceptance of recommended vaccinations during pregnancy: a cross-sectional study in Southern Italy

**DOI:** 10.3389/fpubh.2023.1132751

**Published:** 2023-05-12

**Authors:** Francesca Licata, Marika Romeo, Concetta Riillo, Gianfranco Di Gennaro, Aida Bianco

**Affiliations:** Department of Health Sciences, School of Medicine, University of Catanzaro “Magna Græcia”, Catanzaro, Italy

**Keywords:** COVID-19 vaccine, influenza, pregnancy, pregnant women, Tdap, vaccination, vaccine-preventable diseases (VPDs)

## Abstract

**Background:**

Vaccine administration is a recommended, safe, and effective measure to protect pregnant women against vaccine-preventable diseases (VPDs). Despite available guidance, maternal immunization rates for vaccination against influenza and with the reduced antigen content tetanus-diphtheria-acellular pertussis vaccine (Tdap) in Italy remain incredibly low. The primary goal of the study was to explore what Italian pregnant women knew about VPDs and immunization during pregnancy and what factors affected their decision to be vaccinated.

**Methods:**

This cross-sectional study took place between October 2021 and April 2022 in the Southern part of Italy. All consecutive pregnant women, from those attending the selected facilities on randomly selected days, were approached to request participation. The inclusion criteria for participation were age ≥18 years, the ability to understand, speak, and read Italian, and being pregnant at any gestational age. The questionnaire, using a combination of checkboxes and free text answers, consisted of 32 items divided into five parts and lasted ~10 min.

**Results:**

The results showed that 61% knew that the influenza vaccine is recommended and 48.7% knew that influenza could be risky during pregnancy; 74.1% wrongly reported that the Measles-Mumps-Rubella (MMR) vaccine is recommended during pregnancy. Seven out of 10 pregnant women believed that strong evidence supported the safety of vaccinations during pregnancy, and more than half (55.6%) thought they were at increased risk of severe illness with COVID-19. Women in the sample believed that vaccines received during pregnancy pose a risk of adverse events to the unborn child with a median value of 6 (IQR 3–9), on a scale ranging from 1 to 10. Similarly, the fear of contracting pertussis and influenza during pregnancy showed a median value of 6 (IQR 3–9) and 5 (IQR 3–8), respectively. Only 21.1% and 36.5% of women received influenza and Tdap vaccines during pregnancy.

**Conclusion:**

Unrealistic risk perception with a negative attitude toward vaccines in pregnancy and a low percentage of vaccinated pregnant women confirm the urgency of training women to make informed choices to increase overall vaccine uptake.

## Introduction

Population-based vaccination plans have strongly impacted the transmission of vaccine-preventable diseases (VPDs) either by achieving community immunity or, in some cases, resulting in the eradication of the disease (1). Many detailed studies are needed to confirm that a vaccine is safe and provides adequate protection before approval. After authorization, a safety monitoring system may be carried out to detect any potential rare or very rare side effects (2, 3). Pregnancy is an important time to protect women through vaccination since both the mother and the child are more vulnerable to different diseases (4–6). Indeed, vaccinating women during pregnancy has two distinct potential benefits. First, immunization safeguards the woman from infections that she may be vulnerable to during pregnancy, and ultimately preserves the fetus from congenital infections and other harmful effects of maternal infection (7). Second, maternal vaccination may be adopted to safeguard the fetus and infant during the first months of life through the transplacental transfer of antibodies during pregnancy or through breast milk postnatally (8).

Some vaccines, such as the reduced antigen content tetanus-diphtheria-acellular pertussis (Tdap) vaccine and the one against influenza, if administered during pregnancy, represent an effective way of acting, supplying immunity for newborns through the vulnerable first few months of life (9). Tdap is administered with the primary purpose of preventing infant pertussis (10). Influenza vaccination is also important for pregnant women in whom the infection is associated with a greater risk of morbidity and mortality in addition to the impact that the infection can have on fetal morbidity, including spontaneous abortion, preterm birth, and low birth weight.

The Advisory Committee on Immunization Practices and some international public health authorities strongly recommend vaccines during pregnancy (11–13). In Italy, vaccination advice during pregnancy is a quite recent undertaking, and it is supported by the recommendations stated by the Ministry of Health in August 2018 (14). According to the National Vaccine Prevention Plan 2017–2019 (15), the following vaccinations are strongly recommended during pregnancy in Italy: influenza vaccine, recommended in any trimester of pregnancy, for women who are pregnant during the influenza season; and pertussis vaccination, administered as Tdap vaccine, from 27th to 36th week, ideally around the 28th, with only one dose during each pregnancy regardless of the interval since the last. Furthermore, as a result of the COVID-19 pandemic, the Italian Society of Obstetrics and Gynecology (16) now recommend the COVID-19 vaccine to pregnant women. This vaccine can be administered in any trimester of pregnancy, at the same time as the influenza or pertussis vaccination; but a period of 4 months between each COVID-19 dose is necessary (17). All these vaccinations are offered free of charge to pregnant women.

Although international and national recommendations are available, maternal immunization rates against influenza and Tdap in Italy remain extremely low (15, 18). Barriers to vaccination would include poor knowledge about the safety of vaccines and/or the severity of the illnesses during or after pregnancy, a short time spent with each pregnant woman by obstetric care providers, and a lack of explicit recommendations to get a vaccine (19–21). This study set out to explore primarily what Italian pregnant women knew about VPDs and immunization during pregnancy and what factors affected their decision to be vaccinated.

## Materials and methods

### Study design and study population

This cross-sectional study took place between October 2021 and April 2022 in the Calabria region, in the southern part of Italy. A multi-stage sampling design was used. First, we selected, by simple random sampling, one regional and district general hospital in each of the three areas of the region (north, central, and south). Regional hospitals have autonomous management and provide highly specialized healthcare, whereas district hospitals provide a high standard but a less complex level of care. The aims and methods of the study were delineated to the management staff by phone, and verbal consent to carry out the survey was obtained. All facilities invited agreed to participate in this survey. The study population included all consecutive pregnant women attending outpatient obstetric and gynecology visits or during inpatient days in the facilities on randomly selected days. The inclusion criteria for participation were age ≥18 years, the ability to understand, speak, and read Italian, and being pregnant at any gestational age. Two trained physicians, not involved in patient care, gave pertinent information about the survey (i.e., background, objectives, and privacy rules) to the pregnant women who had given their consent to participate in the study. Strict confidentiality of the data was maintained throughout the process of data collection, entry, and analysis. Women who declined to sign the informed consent form were excluded from the study. Participants did not perceive any form of payment or incentive for taking part in this investigation.

### Questionnaire design

The research team developed the questionnaire based on a literature review of similar studies (22–27). The questionnaire's comprehensibility, clarity, and ease of administration were evaluated by a pilot test (on 10 pregnant women not included in the final sample). Minor refinements were made based on the feedback received from this phase. The final questionnaire, using a combination of checkboxes and free text answers, consisted of 32 items divided into five parts and lasted ~10 min. The first section of the questionnaire (seven items, including closed-ended items with multiple answers and open-ended items) collected information about the sociodemographic characteristics (i.e., age, education level, marital status, and employment status) and pregnancy (i.e., number, gestational age, and complications during pregnancy). The second section (ten items with multiple answers of “true, false, don't know”) investigated general knowledge of the recommended vaccinations during pregnancy. The third section [(eight items, five items on a 5-point Likert scale, ranging from “strongly disagree” to “strongly agree” and two items on a 10-point Likert scale, ranging from 1 (no fear) to 10 (the highest level of fear)] measured attitudes toward the safety and efficacy of vaccines and the perception of VPDs' risk for themselves and their unborn child. The fourth section (six items with multiple answers and open options) explored adherence to recommended vaccinations and information or advice received during pregnancy. Moreover, women were asked if the father of the unborn child had received the Tdap vaccine during the present pregnancy. The last section (two items, closed-ended with multiple answers and open options) explored the main sources of information and the need to receive additional information about recommended vaccinations during pregnancy. The questionnaire is available as Supplementary material.

The Regional Human Research Ethics Committee (ID No. 275/2021/07/15) approved the study protocol.

### Statistical analysis

All collected variables were summarized using means and standard deviations when normally distributed, and medians and interquartile ranges in cases of deviations from normality. The skewness of the variables was evaluated by the Shapiro–Wilk tests. Categorical variables were expressed in percentages. The knowledge score was calculated by assigning one point for each right response and summing the scores (range 0–10) for each statement. Logistic regression models were developed to explore the role of potential predictors of the following outcomes of interest: having received at least one recommended vaccine during pregnancy (no = 0; yes = 1) (Model 1), having received Tdap vaccine during pregnancy (no = 0; yes = 1) (Model 2), having received influenza vaccine during pregnancy (no = 0; yes = 1) (Model 3), and having received COVID-19 vaccine during pregnancy (no = 0; yes = 1) (Model 4). Eligible women for the Tdap vaccine were those during the 27th through 36th week of pregnancy at the time of interview; eligible women for the influenza vaccine were those at any stage of pregnancy during the flu season; eligible women for COVID-19 vaccine were those on or after the 13th week of pregnancy, according to recommendations at the time of the study. The following independent variables were included in the models to explain the response variables: age (continuous, in years); marital status (single/divorced/widow = 0; married or living with a partner = 1); employment status (unemployed = 0; employed = 1); number of pregnancies (one = 0; more than one = 1); having had any complications during pregnancy (yes = 0; no = 1); education level (primary or secondary school = 0; college degree or higher = 1); knowledge that Tdap vaccine is recommended during pregnancy, that both parents have to receive Tdap vaccine to protect newborns, that influenza vaccine is recommended during pregnancy, that influenza could cause severe illness during pregnancy, that influenza increases the risk of spontaneous abortion, preterm birth, and fetal death, that pregnancy is a risk factor for severe illness with COVID-19 (I do not know/false = 0; true = 1) or, alternatively, the knowledge score about vaccinations in pregnancy (ordinal); belief that strong evidence supports the safety and efficacy of vaccinations during pregnancy, that giving multiple vaccines (e.g., Tdap) poses a risk of adverse events, sometimes life-threatening, to the unborn child; and that pertussis poses a serious risk to newborns not yet vaccinated and that pregnant women are at increased risk of severe illness with COVID-19 (uncertain/strongly disagree/disagree = 0; strongly agree/agree = 1). Moreover, fear of vaccine adverse events for the unborn child and of contracting pertussis and/or influenza during pregnancy (continuous), having received a Tdap and/or influenza vaccination recommendation from HCWs, and the need for further information about vaccination during pregnancy (no = 0; yes = 1) were also explored.

The goodness of fit of the logistic model was assessed by the Hosmer and Lemeshow test and by the visual investigation of the lowess curve that fitted the liner predictor (log-odds) values and the Pearson Standardized residuals. The statistical significance level was fixed at a *p*-value of < 0.05. Adjusted odds ratios (ORs) and 95% confidence intervals (CIs) were calculated. Statistical analysis was developed using the STATA software program, version 16.1 (28).

The dataset was deposited in the Mendeley Data repository (https://doi.org/10.17632/53rchjbxrt.1).

## Results

### Participants' socio-demographic and clinical characteristics

Of the 500 women approached, 421 agreed to participate with a response rate of 84.2%. The average age was 32.4 years (±5.1). Less than half (43%) of the sample held a university degree, just over half (55.8%) were employed, and the majority (93.8%) declared to be married or living with a partner. More than half (51.8%) were pregnant for the first time, 65.8% were in the third trimester of pregnancy, and the majority (81.7%) had no complications during pregnancy. Table 1 shows participant characteristics.

**Table 1:** Characteristics of the study population (421 respondents).

	***N***	**%**	**Mean ±SD**
**Age, in years**			32.4 ± 5.1
**Education level**			
Primary or secondary school	240	57	
College degree or higher	181	43	
**Employment status**			
Unemployed	186	44.2	
Employed	235	55.8	
**Marital status**			
Single/divorced/widow	26	6.2	
Married or living with a partner	395	93.8	
**Number of pregnancies**			
1	218	51.8	
>1	203	48.2	
**Trimester of pregnancy**			
First	20	4.8	
Second	124	29.4	
Third	277	65.8	
**Having complications during pregnancy**			
Yes	77	18.3	
No	344	81.7	

### Respondents' knowledge related to VPDs and vaccinations during pregnancy

Table 2 presents the answers to the statements about vaccinations during pregnancy. The overall median knowledge score was 5 [interquartile range (IQR) 4–7], and just 2.6% of the respondents gave the correct answer to all 10 statements. Three-quarters (74.1%) of the interviewed women wrongly affirmed that the Measles, Mumps, and Rubella (MMR) vaccine is recommended during pregnancy, whereas seven out of 10 (70.1%) were knowledgeable that vaccines help to protect pregnant women and their babies during the first few months of life before they are exposed to childhood vaccination. Moreover, less than half of the sample was aware that both parents have to receive the Tdap vaccine to protect newborns before they are exposed to childhood vaccination (47.3%), that influenza could cause severe illness during pregnancy (48.7%), and that it increases the risk of abortion, preterm birth, and fetal death (44.2%).

**Table 2:** Respondents' knowledge related to VPDs and vaccinations during pregnancy.

**Statements (421 respondents)**	**Correct**	
	***N***	**%**
Vaccines stimulate a response from the immunity system to a virus or bacterium (true)	365	86.7
During pregnancy recommended vaccines are:		
Tdap vaccine (true)	295	70.1
MMR vaccine (false)	109	25.9
Influenza vaccine (true)	257	61
Vaccines are exclusively administered during the third trimester of pregnancy (false)	154	36.6
Vaccines help to protect pregnant women and their babies during the first few months of life (true)	295	70.1
Both parents have to receive Tdap vaccine to protect newborns (true)	199	47.3
Influenza could cause severe illness during pregnancy (true)	205	48.7
Influenza increases the risk of spontaneous abortion, preterm birth, and fetal death (true)	186	44.2
Pregnancy is a risk factor for severe illness with COVID-19 (true)	200	47.5

VPDs, vaccine-preventable diseases; Tdap, reduced antigen content tetanus-diphtheria-acellular pertussis vaccine; MMR, Measles, Mumps, and Rubella.

### Respondents' attitudes toward vaccines during pregnancy

Table 3 displayed respondents' attitudes toward vaccines recommended during pregnancy. In the study population, 68.4% of pregnant women believed that strong evidence supports the safety of vaccinations during pregnancy and more than half (55.6%) thought to be at increased risk of severe illness with COVID-19. With respect to vaccine risk perception, the women believed that vaccines received during pregnancy pose a risk of adverse events to the unborn child, with a median value of 6 (IQR 3–9). Moreover, the fear of contracting pertussis and influenza during pregnancy showed, respectively, a median value of 6 (IQR 3–9) and 5 (IQR 3–8) out of a maximum score of 10.

**Table 3:** Respondents' attitudes toward vaccines during pregnancy.

**Statements (421 respondents)**	**Strongly disagree/Disagree**		**Uncertain**		**Strongly agree/Agree**	
	***N***	**%**	***N***	**%**	***N***	**%**
Strong evidence supports safety of vaccinations during pregnancy	44	10.5	89	21.1	**288**	**68.4**
Strong evidence supports efficacy of vaccinations during pregnancy	46	10.9	85	20.2	**290**	**68.9**
Giving multiple vaccines (i.e., Tdap) during pregnancy poses risk of adverse events to the unborn child	**163**	**38.7**	146	34.7	112	26.6
Pertussis poses serious risk to newborns not yet vaccinated, sometimes life-threatening	26	6.1	111	26.4	**284**	**67.5**
Pregnant women are at increased risk of severe illness with COVID-19	76	18	111	26.4	**234**	**55.6**

The numbers and percentages referring to correct answers are in bold.

Tdap, reduced antigen content tetanus-diphtheria-acellular pertussis vaccine.

### Respondent's acceptance, intentions to receive vaccinations, and factors influencing vaccination uptake during pregnancy

Table 4 shows the uptake of the recommended vaccinations during pregnancy. Among eligible pregnant women for the Tdap vaccination, just 36.5% reported having received it. With regard to influenza vaccination, all respondents were eligible but just a little more than one-fifth (21.1%) affirmed having received the vaccine, and among those eligible almost two-fifths (38.7%) of women received COVID-19 vaccination. More than one-third of the sample had neither been vaccinated nor intended to get vaccinations during pregnancy (38.5%), and the most common reasons cited for not getting themselves vaccinated were fear of side effects (60.3%) and lack of recommendation by HCWs (41%). Additional cited arguments were preferring natural immunity (18.6%), considering vaccines not effective (14.9%), and being anti-vax regardless of pregnancy (9.3%). On the other hand, the strongest factors that had driven pregnant women to get or intend to get vaccinated were protecting the newborn (78.8%), having received recommendations from HCWs (58.7%), usually receiving recommended vaccines (44%), and belief that vaccines can prevent severe illness (35.1%). The results of the multiple logistic regression analysis (Model 1 in Table 5) indicated that the strongest predictors of having received at least one recommended vaccine during pregnancy were having received a Tdap vaccination recommendation by HCWs (OR: 4.91; 95% CI: 2.41–9.99; *p* < 0.001) and lower levels of fear that vaccines received during pregnancy pose the risk of adverse events to the unborn child (OR: 0.81; 95% CI: 0.73–0.89; *p* < 0.001). Moreover, with every one-point increase in the knowledge score (OR: 1.21; 95% CI: 1.05–1.38; *p* = 0.007), the odds of having received vaccination during pregnancy resulted in a 21% increase. Believing that strong evidence supports vaccine safety (OR: 4.23; 23% CI: 1.34–13.38; *p* = 0.014) was significantly more likely in women who have received vaccinations during pregnancy. The results of the logistic regression analysis (Model 2 in Table 5) also indicated that having received the Tdap vaccine during pregnancy was more likely among women who had received a Tdap vaccination recommendation by HCWs (OR: 10.37; 95% CI: 3.90–27.58; *p* < 0.001) and among those who are knowledgeable that Tdap vaccine is recommended during pregnancy (OR: 10.13; 95% CI: 3.43–29.87; *p* < 0.001). Lower odds of having received Tdap vaccination were shown as increased levels of fear that vaccines received during pregnancy pose a risk of adverse events to the unborn child (OR: 0.76; CI: 0.68–0.85; *p* < 0.001).

**Table 4:** Women' acceptance of vaccines during pregnancy.

**Statements**	**Yes**	
	***N***	**%**
Having received influenza vaccine during pregnancy (421)	89	21.1
Having received Tdap vaccine during pregnancy (304)^a^	111	36.5
Having received COVID-19 vaccine during pregnancy (406)^b^	157	38.7

^a^Eligible participants were those on or after the 27th week of pregnancy.

^b^Eligible participants were those on or after the 13th week of pregnancy, according to recommendations at the time of the study.

Tdap, reduced antigen content tetanus-diphtheria-acellular pertussis vaccine.

**Table 5:** Results of the regression model for potential determinants of the outcomes of interest.

**Variables**	**OR**	**95% CI**	***p***
**Model 1. Outcome: having received at least one recommended vaccine during pregnancy Log-likelihood** = −**180.14829; Prob** > **chi2**<**0.001; Obs** = **421**			
Having received Tdap vaccination recommendation by HCWs			
No^*^	1.00		
Yes	4.91	2.41–9.99	< 0.001
Fear that vaccines received during pregnancy pose risk of adverse events to the unborn child, continuous	0.81	0.73–0.89	< 0.001
Knowledge score about vaccinations in pregnancy, ordinal	1.21	1.05–1.38	0.007
Belief that strong evidence supports safety of vaccinations during pregnancy			
Uncertain/strongly disagree/disagree^*^	1.00		
Strongly agree/agree	4.23	1.34–13.38	0.014
Age in years, continuous	1.05	0.99–1.11	0.079
Having received influenza vaccination recommendation during present or last season by HCWs			
No^*^	1.00		
Yes	1.65	0.81–3.31	0.162
Belief that strong evidence supports efficacy of vaccinations during pregnancy			
Uncertain/strongly disagree/disagree^*^	1.00		
Strongly agree/agree	0.53	0.17–1.72	0.294
Education level			
Primary or secondary school^*^	1.00		
College degree or higher	0.65	0.35–1.20	0.169
Fear of contracting pertussis during pregnancy, continuous	1.06	0.97–1.17	0.211
Marital status			
Single/divorced/widow^*^	1.00		
Married or living with a partner	1.61	0.57–4.53	0.370
Fear of contracting influenza during pregnancy, continuous	1.03	0.94–1.14	0.511
Having complications during pregnancy			
Yes^*^	1.00		
No	0.88	0.01–0.46	0.739
Number of pregnancies			
1^*^	1.00		
>1	0.91	0.52–1.57	0.733
Employment status			
Unemployed^*^	1.00		
Employed	1.23	0.69–2.21	0.480
**Model 2. Outcome: having received Tdap vaccine during pregnancy Log-likelihood** = −**117.22296; Prob** > **chi2**<**0.001; Obs** = **304**			
Having received Tdap vaccination recommendation by HCWs			
No^*^	1.00		
Yes	10.37	3.90–27.58	< 0.001
Tdap vaccine is recommended during pregnancy			
I don't know/false^*^	1.00		
True	10.13	3.43–29.87	< 0.001
Fear that vaccines received during pregnancy pose risk of adverse events to the unborn child, continuous	0.76	0.68–0.85	< 0.001
Need for further information about vaccination during pregnancy			
No^*^	1.00		
Yes	0.57	0.29–1.12	0.104
Giving multiple vaccine (i.e. Tdap) during pregnancy poses risk of adverse events to the unborn child			
Uncertain/strongly agree/agree^*^	1.00		
Strongly disagree/disagree	1.57	0.78–3.17	0.210
Number of pregnancies			
1^*^	1.00		
>1	0.65	0.32–1.28	0.213
Education level			
Primary or secondary school^*^	1.00		
College degree or higher	1.51	0.74–3.11	0.257
Having complications during pregnancy			
Yes^*^	1.00		
No	0.64	0.28–1.48	0.300
Age in years, continuous	1.03	0.96–1.10	0.428
Marital status			
Single/divorced/widow^*^	1.00		
Married or living with a partner	1.66	0.30–9.10	0.559
Fear of contracting pertussis during pregnancy, continuous	1.03	0.92–1.14	0.615
Employment status			
Unemployed^*^	1.00		
Employed	0.83	0.39–1.76	0.634
Pertussis poses serious risk to newborns not yet vaccinated, sometimes life–threatening			
Uncertain/strongly disagree/disagree^*^	1.00		
Strongly agree/agree	0.93	0.40–2.16	0.862
Both parents have to receive Tdap vaccine to protect newborns			
I don't know/false^*^	1.00		
True	1.04	0.51–2.11	0.910
**Model 3. Outcome: having received influenza vaccine during pregnancy Log–likelihood** = −**130.80494; Prob** > **chi2**<**0.001; Obs** = **421**			
Having received influenza vaccination recommendation during present or last season by HCWs			
No^*^	1.00		
Yes	19.69	5.73–67.72	< 0.001
Fear that vaccines received during pregnancy pose risk of adverse events to the unborn child, continuous	0.76	0.68–0.86	< 0.001
Need for further information about vaccination during pregnancy			
No^*^	1.00		
Yes	0.42	0.22-0.79	0.008
Education level			
Primary or secondary school^*^	1.00		
College degree or higher	2.19	1.10–4.35	0.025
Influenza vaccine is recommended during pregnancy			
I don't know/false^*^	1.00		
True	2.47	1.03–5.89	0.042
Marital status			
Single/divorced/widow^*^	1.00		
Married or living with a partner	0.31	0.09–1.05	0.060
Fear of contracting influenza during pregnancy, continuous	1.09	0.97–1.22	0.134
Influenza could cause severe illness during pregnancy			
I don't know/false^*^	1.00		
True	1.70	0.59–4.91	0.322
Having complications during pregnancy			
Yes^*^	1.00		
No	1.21	0.51–2.91	0.658
Age in years, continuous	0.99	0.92–1.05	0.666
Influenza increases the risk of spontaneous abortion, preterm birth, and fetal death			
I don't know/false^*^	1.00		
True	0.90	0.33–2.45	0.836
Employment status			
Unemployed^*^	1.00		
Employed	0.98	0.49–1.99	0.965
Number of pregnancies			
1^*^	1.00		
>1	0.99	0.51–1.94	0.991
**Model 4. Outcome: having received COVID**−**19 vaccination during pregnancy Log–likelihood** = −**251.1856; Prob** > **chi2**<**0.001; Obs** = **406**			
Fear that vaccines received during pregnancy pose risk of adverse events to the unborn child, continuous	0.85	0.80–0.92	< 0.001
Pregnant women are at increased risk of severe illness with COVID-19			
Uncertain/strongly disagree/disagree^*^	1.00		
Strongly agree/agree	1.97	1.19–3.28	0.009
Marital status			
Single/divorced/widow^*^	1.00		
Married or living with a partner	1.83	0.71–4.72	0.214
Age in years, continuous	1.01	0.97–1.06	0.561
Having complications during pregnancy			
Yes^*^	1.00		
No	1.15	0.66–2.01	0.621
Need for further information about vaccination during pregnancy			
No^*^	1.00		
Yes	1.11	0.72–1.71	0.642
Employment status			
Unemployed^*^	1.00		
Employed	1.11	0.68–1.80	0.671
Pregnancy is a risk factor for severe illness with COVID-19			
I don't know/false^*^	1.00		
True	0.94	0.57–1.55	0.811
Number of pregnancies			
1^*^	1.00		
>1	0.98	0.63–1.53	0.928
Education level			
Primary or secondary school^*^	1.00		
College degree or higher	1.02	0.63–1.65	0.938

^*^Reference category.

HCWs, healthcare workers; Tdap, reduced antigen content tetanus-diphtheria-acellular pertussis vaccine.

Having received an influenza vaccination recommendation during the present or previous season by an HCW (OR: 19.69; 95% CI: 5.73–67.72; *p* < 0.001) increased almost by 20 folds the odds of having received the influenza vaccination (Model 3 in Table 5). Furthermore, knowing that the influenza vaccine is recommended during pregnancy (OR: 2.47; 95% CI: 1.03–5.89; *p* = 0.042) and having a college degree or higher education level (OR: 2.19; 95% CI: 1.10–4.35; *p* = 0.025) were positively associated with having received the influenza vaccine. On the other hand, the odds of having received the influenza vaccine during pregnancy decreased by 24% for a one-point increase in the fear that vaccines received during pregnancy pose a risk of adverse events to the unborn child (OR: 0.76; 95% CI: 0.68–0.86; *p* < 0.001). Otherwise, a negative association was also shown for participants who needed further information about vaccinations during pregnancy (OR: 0.42; 95% CI: 0.22–0.79; *p* = 0.008).

As shown in Model 4, respondents who believed that pregnant women are at increased risk of severe illness with COVID-19 (OR: 1.97; 95% CI: 1.19–3.28; *p* = 0.009) and those with lower levels of fear about the risk of vaccine adverse events in the unborn child (OR: 0.85; 95% CI: 0.80-0.92; *p* < 0.001) were more likely to have received COVID-19 vaccine during pregnancy (Table 5).

### Respondents' sources of information

Approximately two-thirds of the pregnant women reported receiving information from HCWs about Tdap and influenza vaccination (65.6 and 64.4%, respectively). A slightly low proportion of women received the recommendation of HCWs to get vaccinated against pertussis (62.7%) and influenza (58.9%). Among the sources of information about immunization during pregnancy, respondents most frequently mentioned HCWs (56.2%) and the Internet (15.2%). The participants declared to be most satisfied with the information provided by obstetricians-gynecologists (OBs), general practitioners (GPs), midwives, and the Internet (81, 43.7, 32.5, and 31.4% respectively). Just over half (51.5%) of the respondents reported that they needed more information about vaccination during pregnancy.

## Discussion

The present research provides up-to-date data about drivers of vaccine hesitancy that is useful to address reluctance about vaccines and to prompt an open dialogue when gaps in immunization status among pregnant women are recognized. Indeed, the current increase in hesitancy about the safety and efficacy of vaccines calls for urgent commitment to discuss the evidence-based benefits of vaccination during pregnancy (29).

Important findings of the present study can be enucleated in three main areas: lack of knowledge, unrealistic risk perception with negative attitudes toward vaccines, and low uptake of vaccines within the Italian context. First, results showed a low level of knowledge related to which vaccines are commonly recommended during pregnancy, with almost 40% of the sample not knowing that the influenza vaccine is one of the recommended vaccines during pregnancy and just one-quarter of the responders know that the MMR vaccine is not recommended in pregnant women. Moreover, half of the responders were not knowledgeable that influenza and COVID-19 infection can cause severe illness during pregnancy and that influenza increases the risk of spontaneous abortion, preterm birth, and fetal death, hence underestimating the risks and bad outcomes of these diseases. It has been demonstrated that appropriate knowledge of the latter is a key determinant of health behavior (30). Results corroborate findings from previous studies (31, 32) and thus highlight not a fully satisfactory level of information within the target population. Moreover, a lack of knowledge and wrong information might lead to a potentially missed opportunity of getting vaccinated or of getting vaccinated during the recommended time frame to give mothers and babies the highest levels of protection. Hence, immunization given at the appropriate time allows the expecting mother to create enough antibodies and the subsequent placental transfer of neutralizing immunoglobulin G (IgG) antibodies and/or secretory immunoglobulin A (IgA) antibodies in the mother's breast milk (33).

Additionally, to shed light on which factors may contribute to women's vaccine hesitancy, attitudes toward vaccines and risk perception have been evaluated and it is worth noting that the aforementioned poor knowledge shares its responsibility for vaccine hesitancy with negative attitudes evident within the research sample. Concern about vaccine safety was an issue in the sample. The tendency to associate serious side effects, to both the mother and the fetus, with vaccines and the underestimation of risks of severe illness during pregnancy, contributed to a better understanding of the phenomenon of vaccine hesitancy from the respondents' point of view. Indeed, it is plausible to affirm that having similar levels of fear of contracting VPDs during pregnancy compared to the concern of serious vaccine-related side effects leads to the same outcome: reluctance in getting vaccinated. Indubitably, the unrealistic perception of risk can be linked to attitudes, experiences, and any worries or obstacles that might influence the choice to vaccinate (34, 35). Therefore, beliefs about the importance of vaccines (36), trust in the information received (37), and realistic risk perception, all seem to play an important role in increasing vaccination uptake during pregnancy. With that said, the results of the multivariate logistic regression analysis corroborate the hypothesis that recommendation by HCWs plays a decisive role in increasing the probability of women getting vaccinated during pregnancy. These findings represent an important insight considering that the National Vaccine Prevention Plan (15) strongly recommends Tdap and influenza vaccines during pregnancy, to be offered actively and free of charge. This raises the question of whether filling up this knowledge gap with more information would be the real key point to overcoming vaccine hesitancy among pregnant women. A systematic review (38) showed how different interventions, such as informing and educating the population, had a significant impact in increasing vaccination coverage. However, vaccine hesitancy remains a critical point (39, 40).

Hence, the third important insight of our research suggests a more specific way of thinking about public sensibilization strategies, since the worrying levels of vaccine coverage during pregnancy remain a public health concern in Italy (41). The essential role of HCWs in informing individuals about the efficacy and safety of immunization has been reported in the literature (42, 43). If a pregnant woman declines vaccination, HCWs (i.e., family physicians, obstetricians and gynecologists, and nurse-midwives) have the responsibility to inquire about her reasons, reintroduce the discussion, and offer the immunization at the next visit. While ensuring the availability of information about maternal immunization and timely recommendation by HCWs, strategies should also put focus on the multiple ways used to address doubts and concerns among pregnant women (44). Considering that the Internet, mass media, and peer network represent the other sources of information women reported using, attention to and the removal of misinformation from these channels could prevent exposure to erroneous data (45). Using strategies that target multiple layers, such as individual, family, and society, it would be then possible to obtain a change in the health-seeking behavior paradigm (46). It is well known that individuals tend to prefer information that confirms their beliefs (confirmation bias) and they are more prone to accept what family members believe, rather than questioning their knowledge (47). Not just that, vaccine hesitancy is a complex and context-dependent phenomenon (48). Therefore, a one-size-fits-all approach is unlikely to be successful. Adopting instead a more tailored type of communication, based on specific concerns, would be more productive and less confrontational. Evidence-based recommendations on intervention strategies to increase vaccination uptake are provided by research (49). Findings include methods for boosting community vaccination demand through systems for recalling individuals, immunization records, and reminders, as well as methods for enhancing the accessibility of vaccination services (e.g., home visits and prenatal vaccination programs) (49, 50). The potential for implementing such interventions in healthcare systems is particularly high. In particular, web-based interventions targeting pregnant women have resulted in higher vaccine uptake (50) and offer a low-cost method of providing knowledge about vaccines. Sharing information and simply providing women with recommendations may not be enough to build on the principle of informed decision-making (51). Communication should be done in a respectful and non-judgmental way, providing a supportive environment for decision-making (52). A lot of effort has indeed been put into reducing vaccine hesitancy during this time, but what appears important here also is the need for the empowerment of pregnant women to allow them to make informed decisions rather than just be compliant with the physician's recommendation.

## Limitations

In the interpretation of the findings from this study, some limitations should be acknowledged. Foremost, one limitation attains to the cross-sectional design of the study, not allowing to conclude causality about the observed associations. Second, the vaccination uptake was self-reported, and it may be subjected to recall bias resulting in an underestimation of the vaccination coverage in pregnant women. However, a retrospective manual medical record review of vaccination uptake was considered impracticable since accurate administrative data were not available, and we believe that recall bias is less likely since recall is limited to pregnancy time. Another limitation is the potential overestimation of positive outcomes: women who were more informed or interested in health topics may have been more willing to participate, but the high response rate minimizes this limitation. Fourth, vaccination uptake data were unavailable for women who did not attend outpatient obstetric and gynecology visits or were not hospitalized and it cannot be excluded that there are differences regarding knowledge, risk perception, and adherence to recommended vaccination between the recruited women and those we were not able to approach. However, to overcome this limit, the sociodemographic characteristics of the participants in some healthcare facilities were compared with those of all the pregnant women referring to that specific healthcare facility, and no considerable differences were found (data not shown). Finally, the study data were collected among pregnant women from only one Italian region which may not represent the entire pregnant population in Italy. However, it is reasonable to suppose that the study's results are at least representative of pregnant women of Southern Italy.

## Conclusion

Unrealistic risk perceptions with negative attitudes toward vaccines in pregnancy and a low percentage of vaccinated pregnant women emerged from the present research. Training women to make informed choices to increase overall vaccine uptake is strongly needed. Improving knowledge and awareness is the first step to building vaccine confidence, and the failure to adequately inform pregnant women increases the skepticism about vaccination and the risks of vaccine hesitancy. Positive messaging around the safety and effectiveness of vaccination could be a good and low-cost way to communicate the usefulness of vaccinations and to strengthen the knowledge about immunization during pregnancy.

## Data availability statement

The datasets presented in this study can be found in online repositories. The names of the repository and accession number can be found below: Mendeley Data repository, http://doi.org/10.17632/wg2kmmm3rz.1.

## Ethics statement

The study was reviewed and approved by the Regional Human Research Ethics Committee (ID No. 275/2021/07/15). The participants provided their written informed consent to participate in this study.

## Author contributions

MR, FL, and GDG participated in the conceptualization and design of the study and contributed to the data analysis and interpretation. MR collected the data. FL and CR contributed to the data collection. MR, FL, and CR contributed to the preparation of the first draft of the manuscript. AB, the principal investigator, designed the study, coordinated and supervised data collection, was responsible for the statistical analysis and interpretation, and wrote the final article. All the authors have given final approval of the version to be published and agreed to be accountable for all aspects of the work.
