# Supplementary material for: Acceptance of recommended vaccinations during pregnancy: a cross-sectional study in Southern Italy
Source: Front Public Health. 2023 May 12;11:1132751. doi: 10.3389/fpubh.2023.1132751 (PMC10213683; doi:10.3389/fpubh.2023.1132751)
Supplement: Supplementary file 1 [file Presentation_1.pdf]

University "Magna Græcia" of Catanzaro
Department of Health Sciences

ACCEPTANCE OF RECOMMENDED VACCINATIONS DURING PREGNANCY

The purposes of the study were to investigate the knowledge and attitudes towards vaccinations during pregnancy, and what factors influence the decision of pregnant women to be vaccinated. According to General Data Protection Regulation (EU) 2016/679, data will be analyzed only in an aggregate way and there will be no nominal data spreading. Precautions have been taken in order to guarantee confidentiality of gathered data and anonymity of respondents, so you can feel completely free to respond.

Section A. Socio-demographic and anamnestic characteristics

The questions in this section aim to acquire general information. Please note that anonymity will be fully maintained.

A1. How old were you on your last birthday? _______

A2. What is your highest level of education?

☐Primary school ☐Secondary school ☐College degree or higher

A3. What is your marital status?

☐Single ☐Married ☐Living with a partner ☐Divorced ☐Widow ☐Other, specify _________

A4. What is your employment status? ☐Unemployed ☐Employed

A5. How many pregnancies have you had? (Including the actual one) _______

A6. What is your gestational age? (Including the actual week) ________

A7. Did your pregnancy proceed without any complications? ☐Yes ☐No

Section B. Knowledge about vaccinations

This section is designed to gather information about knowledge regarding vaccinations.

	True	False	I don't know
**B.1 Vaccines stimulate a response from the immunity system to a virus or bacterium**	☐	☐	☐
**B.2 Reduced antigen content tetanus-diphtheria-acellular pertussis vaccine is recommended during pregnancy**	☐	☐	☐
**B.3 Measles, mumps, and rubella vaccine is recommended during pregnancy**	☐	☐	☐
**B.4 Influenza vaccine is recommended during pregnancy**	☐	☐	☐
**B.5 Vaccines are exclusively administered during the third trimester of pregnancy**	☐	☐	☐
**B.6 Vaccines help to protect pregnant women and their babies during the first few months of life**	☐	☐	☐
**B.7 Both parents must receive reduced antigen content tetanus-diphtheria-acellular pertussis vaccine to protect newborns**	☐	☐	☐
**B.8 Influenza could cause severe illness during pregnancy**	☐	☐	☐
**B.9 Influenza increases the risk of spontaneous abortion, preterm birth, and fetal death**	☐	☐	☐
**B.10 Pregnancy is a risk factor for severe illness with COVID-19**	☐	☐	☐

Section C. Risk perception related to vaccinations during pregnancys

This section is designed to gather information about your attitudes towards vaccinations and vaccine-preventable diseases.

	Strongly disagree	Disagree	Uncertain	Agree	Strongly agree
**C.1 Strong evidence supports safety of vaccinations during pregnancy**	☐	☐	☐	☐	☐
**C.2 Strong evidence supports efficacy of vaccinations during pregnancy**	☐	☐	☐	☐	☐
**C.3 Giving multiple vaccines (i.e., reduced antigen content tetanus-diphtheria-acellular pertussis vaccine) during pregnancy poses risk of adverse events to the unborn child**	☐	☐	☐	☐	☐
**C.4 Pertussis poses serious risk to newborns not yet vaccinated, sometimes life-threatening**	☐	☐	☐	☐	☐
**C.5 Pregnant women are at increased risk of severe illness with COVID-19**	☐	☐	☐	☐	☐

C.6 How would you rate your fear level of contracting influenza during pregnancy?

☐1 ☐2 ☐3 ☐4 ☐5 ☐6 ☐7 ☐8 ☐9 ☐10

No fear **Highest level of fear**

C.7 How would you rate your fear level of contracting pertussis during pregnancy?

☐1 ☐2 ☐3 ☐4 ☐5 ☐6 ☐7 ☐8 ☐9 ☐10

No fear **Highest level of fear**

C.8 How much have you worried about vaccines received during pregnancy and their potential adverse event for the unborn child?

☐1 ☐2 ☐3 ☐4 ☐5 ☐6 ☐7 ☐8 ☐9 ☐10

No fear **Highest level of fear**

Section D. Women's acceptance of vaccines during pregnancy

Below are some questions about your personal experiences with vaccinations.

	Yes	No
**D.1 Have you received information about reduced antigen content tetanus-diphtheria-acellular pertussis vaccine from HCWs?**	☐	☐
**D.2 Have you received information about influenza vaccine from HCWs?**	☐	☐
**D.3 Have you received recommendation to get vaccinated against pertussis from HCWs?**	☐	☐
**D.4 Have you received recommendation to get vaccinated against influenza from HCWs?**	☐	☐

D.5 Did you get vaccinated/are you willing to get vaccinated against influenza and/or pertussis during pregnancy?

☐Yes, against influenza
☐Yes, against pertussis
☐Yes, against both
☐No→ *Skip to question D.7*

D.6 Could you specify the reasons for wanting to get vaccinated during pregnancy? (multiple responses are allowed)

☐I want to protect my baby
☐HCWs recommended vaccines
☐I usually get recommended vaccines
☐Vaccines are able to prevent severe illness
☐Other, specify______
→ *Skip to question D.8*

D.7 Could you specify the reasons for not wanting to get vaccinated during pregnancy? (multiple responses are allowed)

☐Fear of side effects
☐Fear of injections
☐Vaccines are not effective
☐HCWs did not recommend vaccinations
☐Preferring natural immunity

☐Vaccination centres are too far
☐Vaccination centres' opening times
☐I could not keep the appointment/I forgot my appointment
☐Waiting times for vaccination
☐I am anti-vax regardless of pregnancy
☐Other, specify________

D.8 Did the father of the unborn child get vaccinated against pertussis during your current pregnancy?

☐Yes
☐No

Section E. Sources of information

E.1 From which of the following sources did you acquire information about recommended vaccinations during pregnancy? (multiple answers are allowed). Please, rate your level of satisfaction.

	No satisfied	Not very satisfied	Uncertain	Quite satisfied	Very satisfied
☐ Obstetrician-gynecologist	☐	☐	☐	☐	☐
☐ General Practitioner	☐	☐	☐	☐	☐
☐ Midwife	☐	☐	☐	☐	☐
☐ Friends/relatives	☐	☐	☐	☐	☐
☐ Internet	☐	☐	☐	☐	☐
☐ Mass-media	☐	☐	☐	☐	☐
☐ Other, specify______________	☐	☐	☐	☐	☐

E.2 Do you need further information about vaccinations during pregnancy? ☐Yes ☐No

The questionnaire is finished, if you would like to add something more, please write it down in the space below.

Thank you for your valuable collaboration!
